# Identification of Potential Crucial Genes Associated With the Pathogenesis and Prognosis of Endometrial Cancer

**DOI:** 10.3389/fgene.2019.00373

**Published:** 2019-04-26

**Authors:** Li Liu, Jiajing Lin, Hongying He

**Affiliations:** Department of Obstetrics and Gynecology, Liuzhou Worker’s Hospital, Fourth Affiliated Hospital of Guangxi Medical University, Liuzhou, China

**Keywords:** endometrial cancer, bioinformatics, prognosis, biomarker, GEO, TCGA

## Abstract

**Background and Objective:**

Endometrial cancer (EC) is a common gynecological malignancy worldwide. Despite advances in the development of strategies for treating EC, prognosis of the disease remains unsatisfactory, especially for advanced EC. The aim of this study was to identify novel genes that can be used as potential biomarkers for identifying the prognosis of EC and to construct a novel risk stratification using these genes.

**Methods and Results:**

An mRNA sequencing dataset, corresponding survival data and expression profiling of an array of EC patients were obtained from The Cancer Genome Atlas and Gene Expression Omnibus, respectively. Common differentially expressed genes (DEGs) were identified based on sequencing and expression as given in the profiling dataset. Pathway enrichment analysis of the DEGs was performed using the Database for Annotation, Visualization, and Integrated Discovery. The protein–protein interaction network was established using the string online database in order to identify hub genes. Univariate and multivariable Cox regression analyses were used to screen prognostic DEGs and to construct a prognostic signature. Survival analysis based on the prognostic signature was performed on TCGA EC dataset. A total of 255 common DEGs were found and 11 hub genes (TOP2A, CDK1, CCNB1, CCNB2, AURKA, PCNA, CCNA2, BIRC5, NDC80, CDC20, and BUB1BA) that may be closely related to the pathogenesis of EC were identified. A panel of 7 DEG signatures consisting of PHLDA2, GGH, ESPL1, FAM184A, KIAA1644, ESPL1, and TRPM4 were constructed. The signature performed well for prognosis prediction (*p* < 0.001) and time-dependent receiver–operating characteristic (ROC) analysis displayed an area under the curve (AUC) of 0.797, 0.734, 0.729, and 0.647 for 1, 3, 5, and 10-year overall survival (OS) prediction, respectively.

**Conclusion:**

This study identified potential genes that may be involved in the pathophysiology of EC and constructed a novel gene expression signature for EC risk stratification and prognosis prediction.

## Introduction

Endometrial cancer (EC) is a group of epithelial malignancies that occur in the endometrium and is the most common gynecological malignancy in developed countries. It is estimated that the incidence and mortality of EC was 22.2/100,000 and 4.4/100,000, respectively, in Europe and 8.4/100,000, 1.8/100,000, respectively, worldwide in females in 2018 (Ferlay et al., 2018a,b). In China, the incidence and mortality of EC was 6.6/100,000 and 1.54/100,000, respectively, in females in 2014 (Chen et al., 2014). The incidence of EC has increased during recent years based on the population age and population size (Chen et al., 2017; Global Burden of Disease Cancer Collaboration, Fitzmaurice et al., 2018). While great advances have been made regarding treatment options available for EC, such as surgical interventions, radiotherapy and chemotherapy, large differences exist in the outcomes for patients with different stages of EC. Early EC patients usually have good prognosis but advanced, recurrent, or metastatic EC patients commonly have a bad outcome, which contributes to an ineffective response to radical surgery for EC (Creasman et al., 2006; Watari et al., 2009; Mcgunigal et al., 2017). Therefore, there is an urgent need to identify new molecules that can be used as diagnostic biological markers, molecular therapeutic targets, and to predict prognosis of EC.

Endometrial cancer development and progression occurs as a result of environmental factors and genetic variation, and shows different pathological and molecular characteristics. Classification of EC has been established based on different systems including clinical, metabolic, and endocrine, histological, and genetic alterations. These characteristics are usually used as a guide for selecting treatment strategies and prognosis assessments for EC patients (Bokhman, 1983; Han et al., 2013; Murali et al., 2014). A few clinical factors and pathological features further determine risk level and the prognosis of EC patients. Risk stratification comprehensive analysis of EC patients based on tumor stage, clinical and biological prognostic factors has been established and utilized (Pecorelli, 2009; Korkmaz et al., 2017). However, many genes and pathways are also associated with risk level and prognosis of EC patients (Stelloo et al., 2015). Along with the development of next-generation sequencing, a large number of differentially expressed genes (DEGs) have been discovered between EC tissue and normal endometrium tissue, which have been applied to characterize EC into four subtypes (Church et al., 2013). Furthermore, a few DEGs can be used as biomarkers for EC risk stratification and prognosis (O’mara et al., 2016; Corrado et al., 2018). However, only a few studies have conducted a comprehensive analysis of DEGs related to risk judgment and prognosis of EC.

The Cancer Genome Atlas (TCGA) and Gene Expression Omnibus (GEO) database contain many high-throughput sequencing and gene expression profile data of many different cancer types at DNA, RNA, protein, and epigenetic levels. These genomic data are publicly available and play an important role in exploring the molecular characteristics of cancer occurrence, recurrence, as well as metastasis and in improving diagnosis and treatment of cancer (Tomczak et al., 2015). In recent years, a new molecular typing of EC has been developed through comprehensive genomic and transcriptomic analysis of ECs using TCGA high-throughput sequencing data, which can greatly contribute to develop a targeted therapy for a specific genetic mutation population (Mcalpine et al., 2018). Additionally, comprehensive analysis of DEGs based on TCGA and GEO data has found new models consisting of many DEGs that have been used for risk stratification and as potential diagnosis and prognosis biomarkers in certain cancers (Zhou et al., 2015; Huang et al., 2017; Liu X. et al., 2018).

In this study, we first identified DEGs through an integrated analysis based on TCGA and GEO gene expression data of EC tissue and normal endometrial tissue. A bioinformatics analysis was used to analyze potential prognosis biomarkers for predicting the survival of EC patients using TCGA datasets. Finally, we constructed a DEG expression-based prognostic signature, which may contribute to the development of risk stratification and prognosis assessment of EC patients.

## Materials and Methods

### Data Source

The gene microarray expression data of GSE63678, including 7 EC tissue samples and 5 normal endometrial tissue samples was downloaded from the Gene Expression Omnibus (GEO) database^1^. The EC dataset containing 551 tumor samples and 35 normal samples, which included raw counts of mRNA expression data and corresponding clinical information, was obtained from The Cancer Genome Atlas (TCGA) dataset^2^. Data in this study was obtained from GEO and TCGA public databases and the acquisition and application method complied with guidelines and policies of each database.

### Differentially Expressed Gene (DEG) Screening

THE GSE63678 expression profile was normalized and analyzed using the limma package of R software. The TCGA EC dataset was normalized and analyzed using the edgR package of R software. The criteria of a false discovery rate (FDR) *p*-value < 0.05 and | logFC| > 1 were applied to screen the DEGs. The DEGs that were overlapping in the GSE63678 and TCGA EC datasets were named as common DEGs and were clustered using the pheatmap package of R software.

### Functional Enrichment Analysis of DEGs

The Database for Annotation, Visualization and Integrated Discovery (DAVID) v6.8^3^ was used to analyze the common DEGs using gene ontology (GO) enrichment analysis to identify the biological processes, molecular functions, cellular components, and signaling pathways associated with these DEGs. A *p*-value of <0.05 was considered as statistically significant.

### Protein–Protein Interaction (PPI) Network and Module Analysis

The potential relationship between the DEGs encoding proteins was analyzed using the STRING database^4^. Visualization of the PPI network was done using Cytoscape software. Genes with the top 10 highest degrees in the PPI network were viewed as hub genes. Module analysis of the PPI network was performed using the Molecular Complex Detection (MCODE) tool of Cytoscape software. Functional enrichment analysis of the modules was carried out using the DAVID database.

### Survival Analysis

In the TCGA EC dataset, patients with a survival time of more than 30 days were used for the survival analysis. The raw count of the DEGs were log2(*x*+1) transformed and Univariate Cox proportional hazards regression analysis was used to identify the potential genes involved in overall survival. DEGs with a *p*-value < 0.05 were subsequently used for multivariate Cox proportional hazards regression analysis to identify prognostic gene markers. In order to further evaluate the relative contribution of these prognostic gene markers to patient survival prediction, these markers were used as the dependent variable to construct the Cox proportional hazards regression model. A risk score model was constructed using a linear combination of these prognostic gene expression markers with the regression coefficient (β) from the multivariate Cox proportional hazards regression analysis. The formula used is as follows: risk score = expression of gene_1_ × β_1_gene_1_ + expression of gene_2_ × β_2_gene_2_ + … expression of gene_n_ × β_n_gene_n_. Patients were divided into a high-risk group and a low-risk group based on the median risk score. The survival analysis between the high-risk group and low-risk group was done using SPSS 20.0. A time-dependent receiver–operating characteristic (ROC) curve was constructed using the survivalROC package of R software to analyze the predictive accuracy of patient overall survival obtained using the risk score model. In addition, comprehensive survival analysis based on the risk score model and EC subgroups, including EC grade, EC histological type and EC stage, were also performed to evaluate the adequacy of the prognostic gene signature for risk stratification and prognostic analysis of different EC subgroups.

### Statistical Analysis

The univariate and multivariate Cox proportional hazards regression analyses were completed using the survival package of R software and SPSS 20.0, respectively. Survival analysis was performed between high-risk group and low-risk group using the Kaplan–Meier method in SPSS 20.0. Completely random two samples t test was used to analyze the statistical difference in the expression of hub genes between tumor samples and normal samples and between prognostic genes in tumor samples and normal samples or between the high-risk group and low-risk group. A *p*-value of <0.05 was considered to be statistically significant.

## Results

### Identification of Differentially Expressed Genes

**FIGURE 1:**
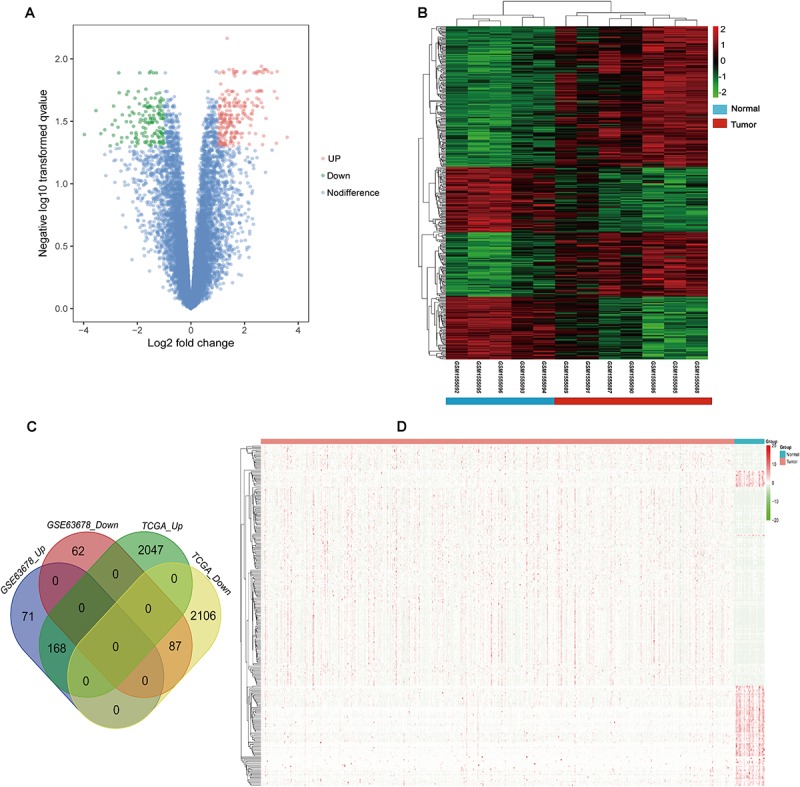
Identification of the DEGs. **(A)** Volcano plot of GSE63678. Red nodes represent DEGs with logFC >1 and *p*-value of <0.05. Green nodes represent DEGs with logFC <–1 and *p*-value of <0.05. **(B)** A heat map of all DEGs of GSE63678. Each column represents a sample and each row represents one gene. The gradual color ranging from green to red represents the gene expression changing from downregulation to upregulation. **(C)** Venn diagrams of common DEGs of GSE63678 and TCGA endometrial cancer (EC) dataset. 71 and 2,047 represent the upregulated DEGs of GSE63678 and TCGA EC dataset, respectively, while 62 and 2,106 represent the downregulated DEGs of GSE63678 and TCGA EC dataset, respectively. 168 represent common upregulated DEGs of GSE63678 and TCGA EC dataset, while 87 represent common downregulated DEGs of GSE63678 and TCGA EC dataset. **(D)** A heat map of the common DEGs in TCGA EC dataset. DEGs, differentially expressed genes; EC, endometrial cancer; TCGA, The Cancer Genome Atlas.

According to the screening criteria, a total of 388 DEGs were found between EC tissue and normal endometrial tissue in GSE63678, which included 239 upregulated and 149 downregulated genes (Supplementary Table S1 and Figure 1A). The hierarchy cluster analysis indicated that DEGs can be distinguished between the two groups based on gene expression (Figure 1B). In addition, 4,410 DEGs were obtained, which consisted of 2,215 upregulated genes and 2,195 downregulated genes in EC tissue when compared with normal endometrial tissue in the TCGA dataset (Supplementary Table S2). Furthermore, 255 common DEGs were identified between the GSE63678 and the TCGA EC dataset which comprised of 168 upregulated genes and 87 downregulated genes (Figure 1C and Supplementary Table S3). Figure 1D shows the cluster analysis of the 255 common DEGs in the TCGA EC dataset.

### Functional and Pathway Enrichment Analysis of the Common DEGs

**FIGURE 2:**
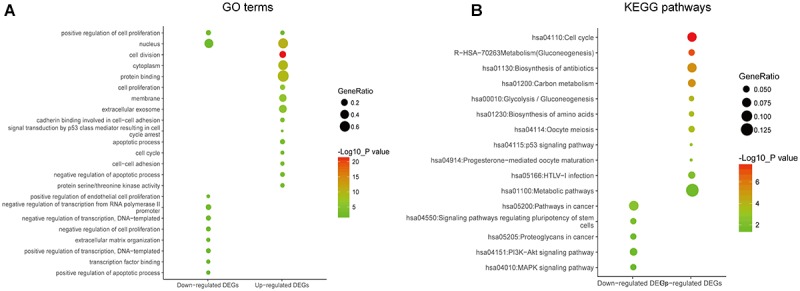
Gene ontology and pathway enrichment analyses of the common DEGs. **(A)** GO enrichment analysis of the common DEGs. The *y*-axis labels represent clustered GO terms. The GeneRatio represents the ratio of the number of genes enriched in one GO term to the number of upregulated or downregulated DEGs. **(B)** KEGG enrichment analysis of the common DEGs. The *y*-axis labels represent clustered KEGG pathways. The GeneRatio represents the ratio of the number of genes enriched in one KEGG pathway to the number of upregulated or downregulated DEGs. GO, gene ontology; DEGs, differentially expressed genes; KEGG, Kyoto Encyclopedia of Genes and Genomes.

Gene ontology and KEGG enrichment analysis were used to explore the biological functions of the DEGs. The upregulated DEGs were mainly associated with cell proliferation, apoptotic process, cell adhesion, and cell cycle, while the downregulated DEGs were mainly enriched in DNA transcription, transcription factor in addition to cell proliferation and apoptosis (Figure 2A and Supplementary Table S4). In the pathway enrichment analysis, metabolic pathways, p53 signaling pathway, and cell cycle were identified for the upregulated DEGs, while the downregulated DEGs were associated with pathways such as PI3K-Akt signaling pathway, MAPK signaling pathway, and signaling pathways regulating pluripotency of stem cells and proteoglycans in cancer (Figure 2B and Supplementary Table S5).

### Protein–Protein Interaction (PPI) Network and Modular Analysis

**FIGURE 3:**
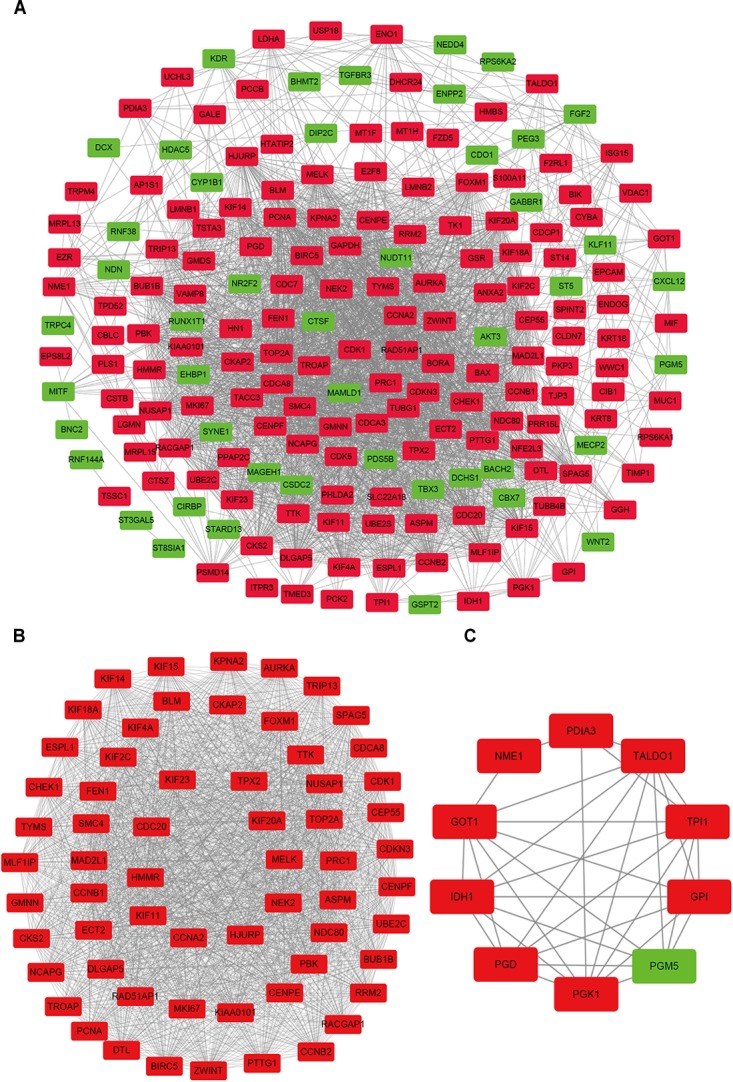
Protein–protein interaction network of common DEGs and module analysis. **(A)** PPI network of proteins encoded by the DEGs, including 194 nodes and 2,581 edges. The yellow circle represents module 2 and the purple circle represents module 1. **(B)** Module 1 consisted of 62 nodes and 1,810 edges. **(C)** Module 2 consisted of 10 nodes and 33 edges. Red nodes and green nodes represent upregulated and downregulated DEGs, respectively. PPI, protein–protein interaction; DEGs, differentially expressed genes.

**FIGURE 4:**
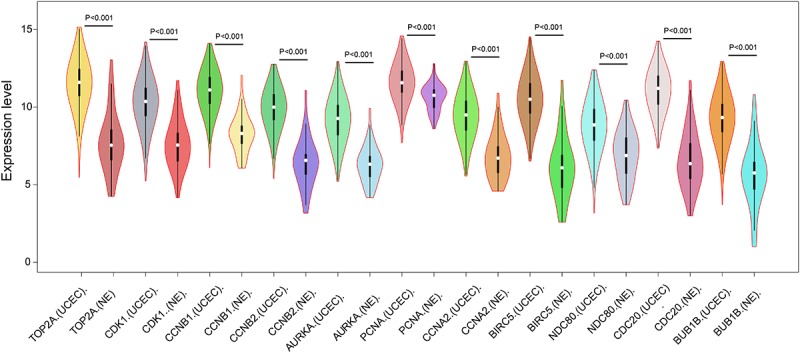
Expression of eleven hub genes in the PPI network in TCGA endometrial cancer dataset between the UCEC group and NE group. The expression value was log2(*X*+1) transformed. Completely randomized two-sample *T*-test was used to calculate the *p*-value. The white dot in each *x*-axis category represents the median. The dark bar on each *x*-axis category shows the interquartile range. The longer gray bar in each *x*-axis category represents the 95% confidence interval. TCGA, The Cancer Genome Atlas; PPI, protein–protein interaction; UCEC, uterine corpus endometrial carcinoma; NE, normal endometrium.

**FIGURE 5:**
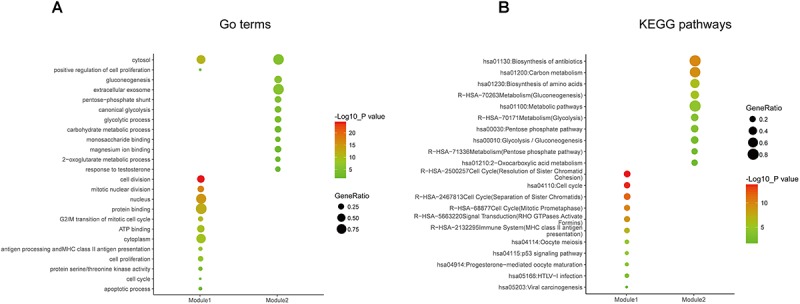
Gene ontology and pathway enrichment analyses of the modules in the PPI network. **(A)** GO enrichment analysis of module 1. The *y*-axis labels represent clustered GO terms. The GeneRatio represents the ratio of the number of genes enriched in one GO term to the number of genes in module 1. **(B)** KEGG pathway enrichment analysis of module 2. The *y*-axis labels represent clustered KEGG pathways. The GeneRatio represents the ratio of the number of genes enriched in one KEGG pathway to the number of genes in module 2. GO, gene ontology; KEGG, Kyoto Encyclopedia of Genes and Genomes; PPI, protein–protein interaction.

In order to reveal the potential relationship between DEGs encoding proteins, a PPI network was constructed based on the SRTING database. A total of 194 proteins obtained from the DEGs and 2,581 edges were included in the PPI network including 46 downregulated genes and 148 upregulated genes (Figure 3A). In the network, nodes with top 10 highest degrees were TOP2A, CDK1, CCNB1, CCNB2, AURKA, PCNA, CCNA2, BIRC5, NDC80, CDC20, and BUB1BA, which were considered as hub genes. According to Cytoscape MCODE soft, two modules were identified in the PPI network. Module 1 contained of 62 nodes and 1,810 edges and module 2 contained 10 nodes and 33 edges (Figure 3B,C). Expression distribution of the 11 hub genes are shown in Figure 4. To our surprise, all 11 hub genes were members of module 1 suggesting that module 1 plays a crucial role in the PPI network. GO terms enrichment analysis suggested that module 1 was mainly involved in diverse cellular activities such as cell division, cell proliferation, apoptotic process, and the cell cycle, while module 2 mainly participates in diverse metabolic processes such as gluconeogenesis, carbohydrate metabolic process, and extracellular exosomes (Figure 5A and Supplementary Table S6). In terms of KEGG enrichment analysis, module 1 was closely related to cell cycle, immune system, p53 signaling pathway and viral carcinogenesis pathways. Module 2 regulated various metabolic pathways such as carbon metabolism and gluconeogenesis (Figure 5B and Supplementary Table S7).

### Survival Analysis

**FIGURE 6:**
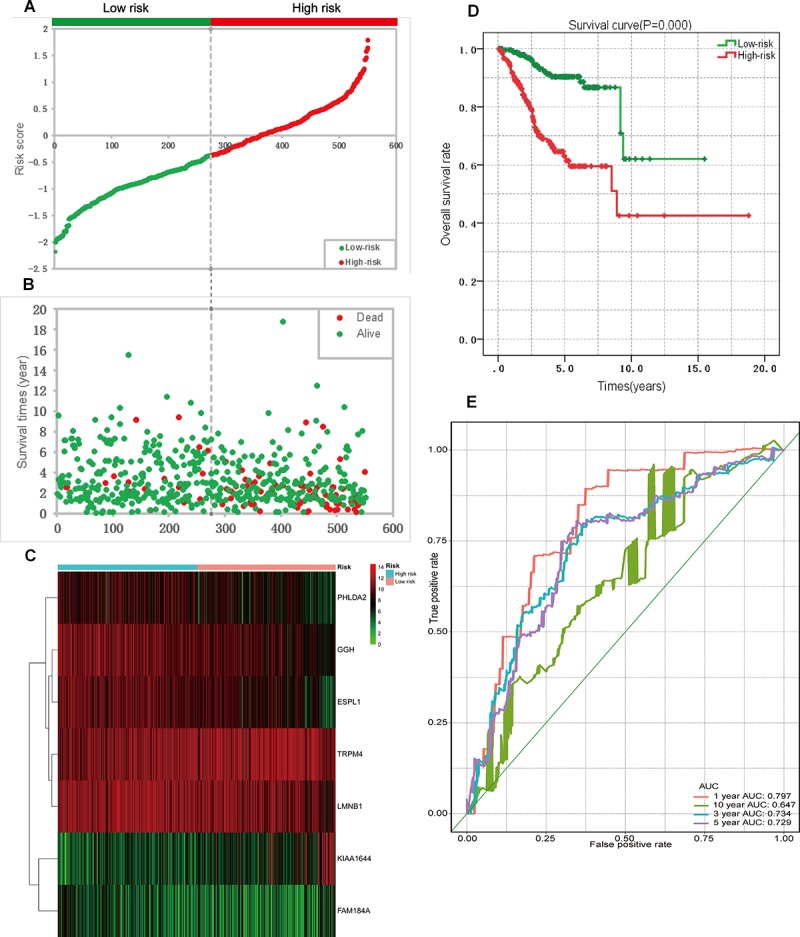
Prognostic analysis based on the seven genes risk score model on TCGA endometrial cancer dataset. **(A)** Patient risk score distribution based on the risk score model. **(B)** Patient survival status distribution of the low-risk group and the high-risk group. **(C)** Heat map of the seven genes that were used to construct the risk score model of the low- and high-risk groups. **(D)** Survival curves for the low- and high-risk groups. **(E)** ROC analysis predicted overall survival using the risk score. TCGA, The Cancer Genome Atlas; ROC, receiver operating characteristic curve.

**FIGURE 7:**
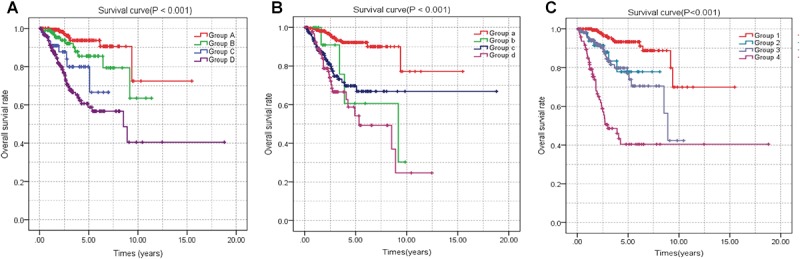
Joint effects analysis of OS stratified by risk score and EC clinical parameters. Joint effects analysis was stratified using the risk score and the following clinical parameters: tumor grade **(A)**, histologic type **(B)**, tumor stage **(C)**. OS, overall survival; EC, endometrial cancer.

**FIGURE 8:**
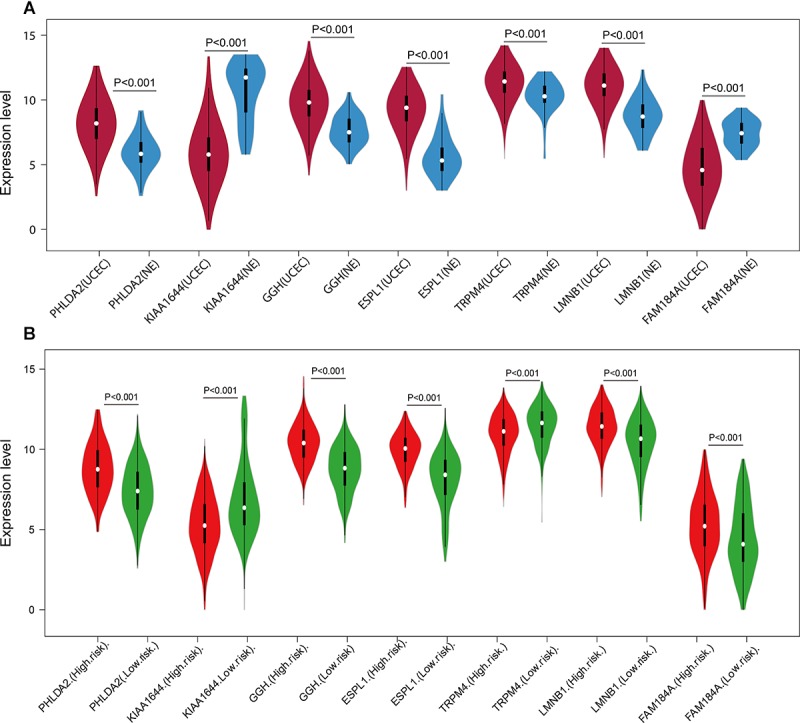
Expression of the seven genes in TCGA endometrial cancer dataset. The expression value was log2(*X*+1) transformed. **(A)** Expression of the seven genes between the UCEC group and NE group in TCGA endometrial cancer dataset. **(B)** Expression of the seven genes of the low- and high-risk groups in TCGA endometrial cancer dataset. The white dot on each *x*-axis category represents the median. The dark bar in each *x*-axis category shows the interquartile range. The longer gray bar in each *x*-axis category represent the 95% confidence interval. TCGA, The Cancer Genome Atlas; UCEC, uterine corpus endometrial carcinoma; NE, normal endometrium.

A univariate Cox regression analysis found that 117 DEGs were associated with patient overall survival (*p* < 0.05) A multivariate Cox proportional hazards regression model constructed the seven DEGs as a prognostic signature for overall survival (*p* < 0.05). These included PHLDA2, KIAA1644, GGH, ESPL1, TRPM4, LMNB1, and FAM184A. Among these genes, PHLDA2, GGH, ESPL1, and FAM184A with a hazard ratio of >1 were regarded as risky prognostic genes, while KIAA1644, ESPL1, TRPM4 with a hazard ratio of <1 were considered as protective prognostic genes (Table 1). According to the risk score model, 276 patients were assigned to the high-risk group and the remaining 275 patients were assigned to the low-risk group. Figure 6A–C presents the risk score state of the TCGA EC dataset. Survival analysis showed that the low-risk group had a better overall survival than the high-risk group (*p* < 0.05) (Figure 6D). The overall survival at 1, 3, and 5 years for low-risk group was 99.6% (95% CI: 1–0.99), 95.6% (95% CI: 0.97–0.90), and 94.2% (95% CI: 0.95–0.86), respectively. Comparatively, overall survival at 1, 3, and 5 years for high-risk group was 92.4% (95% CI: 0.95–0.89), 78.3% (95% CI: 0.78–0.65), and 75.4% (95% CI: 0.71–0.55), respectively. A time-dependent ROC analysis based on the risk score model showed good performance in survival prediction and the area under the ROC curve was 0.797, 0.734, 0.729, and 0.647 for 1, 3, 5, and 10 years, respectively (Figure 6E). Joint effects analysis of the seven-gene signature and EC grade, EC histologic, EC stage also showed a high predictive value for EC patient overall survival (*p* < 0.001) (Figure 7 and Table 2). The overall survival at 1, 3, and 5 years for different EC subgroups based on the seven-gene signature risk stratification model also showed good predictive value (Table 3). The expression value of the seven genes in EC tissue and normal endometrial tissue is shown in Figure 8A, while the expression distribution of these genes in low-risk group and high-risk group is shown in Figure 8B.

**Table 1:** Prognostic value of the seven genes in endometrial cancer patients of the TCGA cohort.

Gene symbol	Univariate analysis		Multivariate analysis		
		
	HR (95% CI)	*p*-value	HR (95% CI)	*p*-value	Coefficient
PHLDA2	1.164 (0.036–268)	0.010	1.203 (1.049–1.378)	0.008	0.185
KIAA1644	0.869 (-0.241– -0.039)	0.006	0.88 (0.788–0.982)	0.022	-0.128
GGH	1.310 (0.138–0.402)	5.99E-05	1.249 (1.038–1.502)	0.018	0.222
ESPL1	1.331 (0.145–0.427)	7.17E-05	1.486 (1.149–1.922)	0.003	0.396
TRPM4	0.857 (-0.292– -0.017)	0.027	0.844 (0.718–0.992)	0.04	-0.17
LMNB1	1.188 (0.020–0.325)	0.027	0.601 (0.439–0.822)	0.001	-0.509
FAM184A	1.111 (0.009–0.201)	0.032	1.153 (1.035–1.285)	0.01	0.142


*TCGA, The Cancer Genome Atlas; CI, confidence interval*.

**Table 2:** Joint effects survival analysis of clinical factors and the DEG signature risk score with OS in EC patients.

Group	Risk score	Variables	Events/total (521)	MST (years)	HR (95%CI)	*P*-value
**Histological grade**						
A	Low risk	G1 + G2	8/164	NA	1	
B	Low risk	G3	11/97	NA	2.198 (0.884-50467)	0.09
C	High risk	G1 + G2	8/47	NA	4.149 (1.553-11.08)	0.005
D	High risk	G3	60/213	8.526	7.115 (3.397-14.901)	<0.001
**Histological type**^a^						
a	Low risk	EEA	14/244	NA	1	
b	Low risk	SEA	4/14	9.175	4.577 (1.495-14.01)	0.008
c	High risk	EEA	32/147	NA	4.645 (2.476-8.714)	<0.001
d	High risk	SEA	31/95	5.326	6.902 (3.662-13.009)	<0.001
**Tumor stage**						
1	Low risk	Stage I + II	12/212	NA	1	
2	Low risk	Stage III + IV	7/49	NA	2.904 (1.138-7.41)	0.026
3	High risk	Stage I + II	26/164	8.907	3.384 (1.702-6.725)	0.001
4	High risk	Stage III + IV	42/96	3.011	11.239 (5.896-21.42)	<0.001


*^*a*^Information of histological type is mixed EEA and SEA for 21 patients. DEGs, differentially expressed genes; OS, overall survival; EC, endometrial cancer; MST, median survival time; HR, hazard ratio; EEA, endometrioid endometrial adenocarcinoma; SEA, serous endometrial adenocarcinoma; CI, confidence interval.*

**Table 3:** 1, 3, and 5-year OS analysis of EC patients based on clinical factors and the DEG signature risk score.

Variables	Risk score	1 year OS (95%CI)	*p*-value	3 year OS (95%CI)	*p*-value	5 year OS (95%CI)	*p*-value
**Histological grade**							
G1 + G2	Low risk	100%		97% (0.96-0.91)		96.3% (0.986-0.89)	
G1 + G2	High risk	93.6% (1.01-0.86)	0.001	85.1% (0.94-0.66)	0.002	85.1% (0.94-0.66)	0.004
G3	Low risk	99% (1.01-0.97)		93.8% (0.98-0.86)		90.7% (0.95-0.76)	
G3	High risk	92% (0.95-0.88)	0.016	77% (0.77-0.62)	<0.001	73.2% (0.68-0.49)	<0.001
**Histological type**							
EEA	Low risk	99.6% (1-0.99)		95.9% (0.98-0.90)		95.1% (0.97-0.88)	
EEA	High risk	91.8% (0.96-0.87)	<0.001	81% (0.83-0.66)	<0.001	78.9% (0.79-0.60)	<0.001
SEA	Low risk	100%		92.9% (1.01-0.74)		78.6% (0.97-0.25)	
SEA	High risk	93.7% (0.99-0.88)	0.341	78.7% (0.78-0.55)	0.155	70.5% (0.70-0.39)	0.43
**Tumor stage**							
Stage I + II	Low risk	100%		97.2% (0.99-0.92)		96.2% (0.98-0.89)	
Stage I + II	High risk	95.7% (0.99-0.92)	0.003	89% (0.91-0.78)	0.001	86.6% (0.87-0.67)	<0.001
Stage III + IV	Low risk	98% (1.02-0.94)		89.8% (0.98-0.78)		85.7% (0.94-0.62)	
Stage III + IV	High risk	86.5% (0.93-0.79)	0.027	60.4% (0.62-0.39)	<0.001	56.2% (0.53-0.27)	<0.001


*^*a*^Information of histological type is mixed EEA and SEA for 21 patients. DEGs, differentially expressed genes; OS, overall survival; EC, endometrial cancer; MST, median survival time; HR, hazard ratio; EEA, endometrioid endometrial adenocarcinoma; SEA, serous endometrial adenocarcinoma; CI, confidence interval.*

## Discussion

In this study, we identified DEGs between EC tissue and normal endometrium based on a GEO expression profile and TCGA high-throughput sequencing, and revealed the hub genes found among the protein-encoding DEGs. We looked for potential biomarkers related to EC prognosis from among the DEGs using univariate and multivariate Cox regression analyses and constructed a prognostic signature based on DEG expression. We found 255 common DEGs and 11 hub genes including TOP2A, CDK1, CCNB1, CCNB2, AURKA, PCNA, CCNA2, BIRC5, NDC80, CDC20, and BUB1BA. We developed a seven-gene signature for prognosis prediction of EC patients, which included the genes PHLDA2, KIAA1644, GGH, ESPL1, TRPM4, LMNB1, and FAM184A. The seven-gene signature displayed good predictive value for OS of EC patients and its subgroups. In summary, these results provide clues for further exploring the pathogenesis of EC and to establish a new risk classification and prognosis assessment model.

Similar to our research, Wu et al. (2019) reported of four important miRNAs that formed a four-miRNA signature that can divide EC patients into a high risk and a low-risk group, with significantly different overall survival according to TCGA EC dataset. A nine-lncRNA signature was also established, which had a good performance in overall survival prediction of endometrioid EC patients based on TCGA dataset (Xu et al., 2018). RNA Sequencing analysis revealed the coexistence of mutations in a three-gene signature that can be viewed as a biomarker for diagnosis of endometrioid EC, while the absence of three-gene signature mutations when TP53 was mutated was found to be diagnostic of serous carcinomas (Cuevas et al., 2019). In our study, a seven-gene signature was developed based on GEO and TCGA EC datasets, which can distinguish between high risk and low risk patients and functions well in predicting the overall survival of EC and its subgroups. In addition, our study showed that metabolic pathways, p53 signaling pathway, and cell cycle were the signaling pathways that were mainly enriched for the upregulated DEGs, while the downregulated DEGs were associated with pathways such as the PI3K-Akt signaling pathway and the MAPK signaling pathway. These results were confirmed by the similar results obtained by Zhang et al. (2016) and Liu et al. (2019).

In the current study, we also found eleven hub genes in the PPI network, indicating that they possibly play an important role in the pathogenesis of EC. Similar to our findings, TOP2A positive EC patients have been found to have shorter overall survival and disease-free survival compared to TOP2A negative EC patients (Lapinska-Szumczyk et al., 2014; Ito et al., 2016). TOP2A heterogeneity was also related to EC stage and metastases. Stage III and IV EC patients and EC patients with EC metastases showed higher TOP2A heterogeneity (Supernat et al., 2014). These results suggest that higher TOP2A levels lead to EC progression and represent a higher degree of malignancy in EC. In other studies, TOP2A was upregulated in cancer tissues when compared with that of adjacent non-cancerous tissues in breast cancer (Wang et al., 2012), renal cell carcinoma (Ye et al., 2018), ovarian cancer (Erriquez et al., 2015), prostate cancer (De Resende et al., 2013), nasopharyngeal carcinoma (Lan et al., 2014), and colon cancer (Zhang et al., 2018). Furthermore, TOP2A overexpression is a positive tumor metastasis marker and a poor biomarker for prognosis. In addition, TOP2A downregulation was found to inhibit the proliferation and migration or invasion of pancreatic and colon cancer cell lines and involved the β-catenin signaling pathway in pancreatic cancer (Pei et al., 2018; Zhang et al., 2018). CCNB1, CCNB2, and CCNA2 are three members of the cyclin family and CDK1, a member of serine-threonine kinases, is a master regulator of cell cycle progression. Furthermore, cell cycle was also enriched significantly in our study for both biological processes and pathways, which indicates cell cycle changes significantly in EC. Consistent with our research, CDK1 and CCNA2 were also found to be overexpressed in EC tissues and cells and were also identified as hub genes in the PPI network (Zhang et al., 2016; Li et al., 2017). At present, there are few studies regarding the role of CCNB1, CCNB2, and CCNA2 in EC. Wang J. et al., 2018 found that CCNA2 expression was high and was positively correlated with histological grades, where a higher expression of CCNA2 was associated with worse differentiation in endometrial adenocarcinoma. CDK1 is a target gene of miR-1271, human paired box 2 and LncRNA ABHD11-AS1 and regulates endometrial carcinoma cell line proliferation, invasion, migration, apoptosis, and other mobility factors (Li et al., 2017; Liu Y. et al., 2018; Wang J. et al., 2018). In vulvar squamous cell carcinoma, elevated levels of CDK1 were found in patients with advanced tumor behaviors and aggressive features (Wang Z. et al., 2015). In addition, a high expression of CDK1 in lung adenocarcinoma patients, epithelial ovarian cancer patients, and colorectal cancer patients was identified as a diagnostic biomarker for poor survival (Sung et al., 2014; Xi et al., 2015; Liu W.T. et al., 2018). AURKA is a human Aurora kinase and is reported to be involved in cell cycle regulation. In a study, AURKA was upregulated in higher tumor grades and was found to be associated with poor histological differentiation in EC (Glover et al., 1995). Furthermore, knockout of AURKA inhibited EC cell line invasion and migration, and improved chemosensitivity to paclitaxel, suggesting that it is a potential therapeutic target in EC (Umene et al., 2015). PCNA is a co-factor of DNA polymerase and is essential for DNA replication. It is also considered to play an important role in the G1 phase to the S phase of the cell cycle (Bolton et al., 1992). PCNA expression was reported to be higher in postmenopausal endometrial carcinoma in comparison to normal postmenopausal endometrium tissue. Furthermore, the expression level of PCNA was found to be related to clinicopathological features and prognosis of EC patients (Hareyama, 1994). Additionally, many studies have demonstrated that it is a poor survival biomarker in osteosarcoma, gastric, and colorectal cancer (Wang et al., 2017; Yin et al., 2017; Zhou et al., 2018). BIRC5 which encodes for survivin protein, is a member of inhibitor of apoptosis gene family and regulates apoptosis, while cell cycle studies suggest that BIRC5 is overexpressed both in EC and in EC cell lines (Pallares et al., 2005; Nabilsi et al., 2009). Furthermore, BIRC5 expression was found to gradually increase from the proliferative endometrium to endometrial hyperplasia to endometrioid adenocarcinoma indicating that it contributes to EC development (Erkanli et al., 2006). In recent years, it was also reported that the high expression of BIRC5 can be used as a biomarker of poor progression free survival (Chuwa et al., 2016). NDC80 is a subunit of the Ndc80 complex and plays an important role in mitotic progression suggesting that NDC80 may be associated with EC through regulation of the cell cycle (Amin et al., 2018). Chen et al. (2011) found that NDC80 was highly expressed in serous adenocarcinomas in comparison with endometrioid adenocarcinomas. However, the expression of NDC80 increased in many cancers such as colon gastric, pancreatic cancer, and osteosarcoma and was associated with poor prognosis. Furthermore, knockdown of NDC80 was found to inhibit cancer cell proliferation and induced apoptosis (Qu et al., 2014; Meng et al., 2015; Xing et al., 2016; Xu et al., 2017). CDC20 is a cell cycle regulating protein. A large number of studies have confirmed that CDC20 is upregulated in solid tumors and promotes cell growth and invasion leading to poor prognosis (Ding et al., 2017; Wang S. et al., 2018). Meanwhile, higher expression of CDC20 was found to be related to a high tumor grade and stage in common malignant tumors including EC (Gayyed et al., 2016). BUB1BA has not been reported in previous studies, and its function remains to be elucidated. All this evidence demonstrates that almost all of the hub genes identified in this study are closely related to tumor development and progression, based on mainly cell cycle regulation. The specific mechanisms by which they regulate EC need to be further investigated.

In addition, we identified 7 pivotal genes involved in EC prognosis and constructed a prognostic gene signature comprising of these genes. Among these, PHLDA2, GGH, ESPL1, and FAM184A are viewed as risky prognostic genes. PHLDA2 is an imprinted gene located on human chromosome 11p15.5. Previous studies have suggested that is a growth suppressor gene and that overexpression of this gene in the placenta leads to growth restricted pregnancies both in humans and in animal models (Jensen et al., 2014). Furthermore, ectopic expression of PHLDA2 results in pregnancy complications possibly by promoting apoptosis and suppressing trophoblast growth (Jin et al., 2016). In cancer, the role of PHLDA2 is controversial. Many studies have shown that PHLDA2 expression is decreased in osteosarcoma tissue and cell lines when compared with controls and that high levels of PHLDA2 is a predictor of good prognosis (Dai et al., 2012; Wang et al., 2016). Additionally, upregulation of PHLDA2 induces osteosarcoma cell apoptosis, inhibited cell growth and tumorigenesis *in vitro* and *in vivo* (Huang et al., 2012; Li et al., 2014). However, PHLDA2 was also found to play oncogenic roles in lung adenocarcinoma (Hsu et al., 2017). In addition, high expression of PHLDA2 has also been observed in triple-negative breast cancer cell lines and pancreatic ductal adenocarcinoma, and represents poor prognosis (Moon et al., 2015). Silencing PHLDA2 reduces cancer cell aggressiveness and proliferation (Idichi et al., 2018). In our study, PHLDA2 expression was upregulated and associated with poor prognosis. These results suggest that the role of PHLDA2 in cancer is complex and further studies are needed to dissect the mechanism of PHLDA2 in EC. GGH is an enzyme involved in folate metabolism. Previous studies have confirmed that GGH is highly expressed in invasive breast cancer and ERG-negative prostate cancer in comparison with adjacent non-cancerous tissues and high GGH levels are related to poor prognosis and unfavorable clinical outcomes (Shubbar et al., 2013; Melling et al., 2017). In oral squamous cell carcinoma, GGH is a member of an 11 gene molecular signature with a worse overall survival maker for patients without nodal metastases (Wang W. et al., 2015). Additionally, it has been identified as a therapeutic target of chemotherapy in multiple cancer types. Lower expression of GGH enhances sensitivity of cancer cells to pemetrexed, 5-fluorouracil, methotrexate, and gemcitabine in colon cancer, advanced pancreatic cancer, and non-small cell lung cancer (Iacopetta et al., 2008; Nakamura et al., 2011; Yoshida et al., 2016). Our results imply that GGH is highly expressed in EC and is a marker of poor prognosis. However, the underlying molecular mechanisms of GGH in EC remain unclear. ESPL1 encoding protein is a protease that cleaves chromosomal cohesin during mitosis. ESPL1 expression has been found to be upregulated in a wide range of cancers (Finetti et al., 2014; Wen et al., 2018) and high expression of ESPL1 is associated with a loss of key tumor suppressor gene P53, which further contributes to the progression of mammary adenocarcinomas (Mukherjee et al., 2014). Nevertheless, it has also been reported that ESPL1 plays an opposite role in gastric adenocarcinoma. Wang D. et al. (2018) showed that ESPL1 levels were lower in gastric adenocarcinoma tissue in comparison with that of adjacent non-cancer tissue and was associated with longer overall survival and a low tumor stage suggesting the dual role of this gene in cancer. ESPL1 expression was found to be increased in our study, however, the clinical significance and functional mechanism of ESPL1 in EC remains to be verified. FAM184A was also found to be increased in the current study and was classified as a risky prognostic gene, but its role in EC has not been reported in previous studies.

Additionally, the three protective prognostic genes identified in this study were TRPM4, LMNB1, and KIAA1644. TRPM4 is a Ca^2+^-activated non-selective cation channel that influences calcium homeostasis. However, it is highly expressed in some cancers and is considered as a risk factor as well as a poor survival factor in prostate cancer and diffuse large B-cell lymphoma (Schinke et al., 2014; Loo et al., 2017). Meanwhile, overexpression of TRPM4 promotes cell proliferation by enhancing the β-catenin signaling pathway and epithelial to mesenchymal transition, migration, and invasion in prostate cancer cell lines (Armisen et al., 2011; Sagredo et al., 2019). In contrast, low expression of TRPM4 was found in colorectal cancer indicating that it may also serve as a protective factor (Sozucan et al., 2015). LMNB1 is an important member of the lamin protein family but its role in cancer is controversial. Its expression is decreased in colon cancer and gastric cancer (Moss et al., 1999), but is increased in prostate cancer, hepatocellular carcinoma, and pancreatic cancer (Sun et al., 2010; Li et al., 2013). Overexpression of LMNB1 indicates lower survival rates both in pancreatic cancer and colon cancer (Li et al., 2013; Izdebska et al., 2018), while upregulation of LMNB1 represents good clinical outcome in breast cancer (Wazir et al., 2013). Furthermore, Fridley et al. (2014) reported that silencing of LMNB1 in cancer cells increases its resistance to cisplatin, suggesting that LMNB1 is beneficial for cancer treatment. Based on the complex role of LMNB1, additional studies are needed to confirm its role in EC. In terms of KIAA1644, little is known about its role and prognostic value in cancer research.

Our study has several limitations. Firstly, our findings are based entirely on public databases using bioinformatics analysis and therefore functional experiments are needed to verify these results. Secondly, the prognostic predictive value of the seven-gene signature is only based on a single cohort with a relatively small sample size and future studies involving larger independent cohorts should be conducted to validate our findings. Additionally, we did not consider common clinical parameters as we only focused on the commonly occurring DEGs, which may have resulted in vital information being ignored.

## Conclusion

In summary, our study identified 255 common DEGs between EC and normal endometrium and identified 11 hub genes and constructed a seven-gene signature that can be used as a good stratified analysis and prognostic prediction biomarker for survival at 1, 3, 5, and 10 years for EC patients. Therefore, our results revealed novel potential molecular therapeutic targets and a new method for EC patient risk stratification assessment and prognostic prediction. Further experimental studies and independent cohort studies are needed to validate these findings.

## Ethics Statement

Data in this study was obtained from GEO and TCGA public database and the acquisition and application method complied with corresponding database guidelines and policies.

## Author Contributions

HH and LL conceived and instructed the work. LL and JL checked the associated database and analyzed the raw data. LL wrote and revised the manuscript. All authors read and approved the final manuscript.

## Conflict of Interest Statement

The authors declare that the research was conducted in the absence of any commercial or financial relationships that could be construed as a potential conflict of interest.

## Footnotes

1 https://www.ncbi.nlm.nih.gov/geo/

2 https://portal.gdc.cancer.gov/

3 https://david.ncifcrf.gov

4 https://string-db.org

## Supplementary Material

TABLE S1 List of differentially expressed genes in GSE63678.

TABLE S2 List of differentially expressed genes between EC tissue and normal endometrial tissue in the TCGA dataset.

TABLE S3 List of common differentially expressed genes both in GSE63678 and the TCGA EC dataset.

TABLE S4 Gene ontology enrichment analysis of the common DEGs both in GSE63678 and the TCGA EC dataset.

TABLE S5 Pathway enrichment analysis of the common DEGs both in GSE63678 and the TCGA EC dataset.

TABLE S6 Gene ontology enrichment analysis of module1 and module2 in PPI network.

TABLE S7 Pathway enrichment analysis of module1 and module2 in PPI network.
